# Identifying High-Priority Profiles for Edentulism and Other Health Outcomes Through Population-Based Clustering

**DOI:** 10.1016/j.focus.2026.100505

**Published:** 2026-05-01

**Authors:** Marjorie A. Rosenberg

**Affiliations:** Department of Risk and Insurance, School of Business, University of Wisconsin-Madison, Madison, Wisconsin

**Keywords:** Cluster analysis, unsupervised machine learning, population health, oral health

## Abstract

**Introduction:**

The aim of this study was to create meaningful clusters of individuals with similar features at the start of the study and to link these clusters to the rate of complete edentulism. These clusters are also linked to death, diabetes, cardiovascular disease, and general distress connecting edentulism to overall health.

**Methods:**

Using the 2019–2022 longitudinal Medical Expenditure Panel Survey, the authors identified baseline risk profiles for adults (aged ≥18 years) through weighted k-modes clustering. To further examine the relationship among these groups, a second-stage hierarchical clustering method was applied to visualize cluster similarities. Input variables were smokers, self-reported health and mental health status, functional limitations, SES, gender, employment, and age. Input variables were measured close to the start of the study. Profiles of clusters were determined on the basis of the modal characteristics of each input variable. External variables (edentulism, death, cardiovascular disease, and diabetes) were assessed over the aggregate 4-year study interval, with Kessler-6 scores for general distress evaluated near to the end of the study.

**Results:**

The second-stage hierarchical analysis identified 3 overall blocks, described as high, medium, and low at-risk, comprising 17 clusters from the first stage. The highest risk block consisted of 5 clusters, all characterized by unfavorable self-reported general health and mental health statuses, yet only 1 cluster identified smoking as a modal characteristic. Within this block, 3 clusters most at-risk had the highest rates of complete edentulism, death, and diabetes, while being among the top 5 clusters with the highest rates of cardiovascular disease and Kessler-6 scores. Although these 3 clusters represented only 8.4% of the total cohort, they accounted for 25.3% of the total edentulous population, 50.0% of all deaths, 22% of all those with diabetes, and 16% of those with cardiovascular disease. The remaining 2 clusters in this block shared similar baseline characteristics but featured a younger age distribution and were more favorable but non-negligible for rates of diabetes or cardiovascular disease than the other 3 clusters.

**Conclusions:**

Clustering provides a pragmatic framework to identify shared characteristics within populations of existing or emerging at-risk groups. By identifying these high-priority groups, healthcare systems can better tailor population health programs to those most in need. Prioritizing initiatives that provide affordable dental care at all ages, alongside oral health literacy programs and investment in preventive services, has the potential to improve oral health and overall health outcomes

## Highlights

• This study developed U.S.-representative risk profiles associated with comorbid outcomes.
• The highest edentulous percentages profiles are associated with perceived healthcare needs.
• Three clusters with 8% of the population account for 25% of the edentulous population and 50% of deaths.
• High-priority groups with imminent and emerging needs can be investigated.

## INTRODUCTION

The loss of all of one’s teeth (complete edentulism) has been noted as a public health concern since the early 1900s.^1^^,^^2^ Reports by the NIH connect edentulism to death and other conditions.^3^^,^^4^

Recent research addressing the relationship between edentulism and death has shown its association with increased all-cause mortality in male veterans.^5^ Complete edentulism rather than missing some teeth is associated with all-cause mortality.^6^ Although thought of as an older person’s condition, Brown (2009) found that complete edentulism prior to age 65 years is also associated with all-cause mortality.^7^ Other work has linked edentulism with cardiovascular disease (CVD),^8^^,^^9^ diabetes,^10^ and dental care.^9^^,^^11^, ^12^, ^13^ Prior work has shown that age, level of education, SES, and being female are significant factors in edentulism among older adults.^14^

In this work, the value of creating clusters of individual risk profiles rather than viewing a list of significant individual characteristics was demonstrated to help develop a practical strategy to improve population health. This work focused on perceived health need to establish a baseline risk and remove the necessity of receiving health care. These clusters were validated on external variables to see whether there is a pattern among them. A 2-stage clustering method was used, where clusters with baseline risk characteristics were created, and then similar clusters were grouped with a hierarchical method. Instead of targeting specific populations, the study included all those aged ≥18 years. This approach uncovers patterns and relationships that are not revealed in regression studies and can be used by health plans and policymakers to target appropriate interventions to improve the health of high-risk individuals or those with potential at-risk profiles.

## METHODS

### Study Sample

The Medical Expenditure Panel Survey (MEPS), an annual survey that is representative of the U.S. civilian, non-institutionalized population, was used. The data are publicly available and were exempt from IRB review.^15^ The data used for this study are representative of individuals aged ≥18 years.

Individuals in Panel 24, a longitudinal cohort, were followed for 4 years (2019–2022 inclusive) with 9 rounds of interviews. Rounds 1 and 2 were in 2019, Round 3 could be either in 2019 or in 2020, Round 4 was in 2020, Round 5 was in either 2020 or 2021, Round 6 was in 2021, Round 7 was in either 2021 or 2022, and Rounds 8 and 9 were in 2022.

Critical to the analysis is the indication of whether a person is completely edentulous, a variable that is not used in the formation of the clusters. A binary variable was defined, indicating whether a person is edentulous at any time across the 4 years of the study, using the question *Have you lost all of your upper and lower natural (permanent) teeth?* from Rounds 3, 5, 7, and 9 for those aged ≥18 years at the beginning of the study. If a person answered *yes* to any of these 4 questions, they were considered completely edentulous. If a person answered *no* to all 4 questions, they were not considered completely edentulous. Only individuals who have answered at least 1 of the edentulous questions were included in this study.

In this study, the baseline risk was established with 8 variables to develop the clusters that are common features seen in the literature relating to edentulism. These variables, used as binary variables, were chosen close to the beginning of the study. Age (18–49 and ≥50 years), gender (male, female), perceived health status (PHS) (excellent/very good; good/fair/poor), mental health status (MHS) (excellent/very good or good/fair/poor), an indicator of unemployment for the entire round, and an indicator of any limitation (work, school, or home) were obtained from the first round of data. Smoking status (smoker, nonsmoker) was obtained from the third round. The income category relative to the federal poverty line (high/medium, low/near, poor/poor) was used from the first calendar year. PHS, MHS, and an indicator of any limitation together can proxy one’s perceived overall health and substitute for diagnosed medical conditions. Using these variables mitigates the need to see a provider and represents how individuals feel overall. Income has been used as a proxy for education as well as for the affordability and access to health insurance and health care.^16^^,^^17^ The goal was to determine whether these variables that define the baseline risk are associated with complete edentulism, death, and other external variables.

Although the data are longitudinal, the study methods do not reflect time-varying variables. As mentioned, edentulism was defined by aggregating 4 different measurements into 1 binary variable that indicates whether or not a person is edentulous. Similarly, death was measured by an indicator variable as to whether or not a person died in the 4-year period. The baseline risk was compared with these 2 variables as well as with other external variables (diabetes, CVD) (combining diagnosed chronic heart disease, angina, myocardial infarction, stroke, and other heart issues, and high blood pressure), and high psychological distress using the Kessler-6 (K6) score in round 8). Diabetes and CVD are measured similarly to edentulousness and death, as being diagnosed in any of the four years of the MEPS study. In this way all of the external variables are a reflection of a person’s cumulative medical history. The Kessler-6 score, measured at the end of the study, captures the state of distress at that time.

Inherent in a longitudinal study is missing data; however, the complex survey design adjusts for data loss to follow-up or non-response. If and when individuals died, left their reporting unit, became institutionalized, or became active-duty military, their future responses were set to “inapplicable”. As the baseline risk is set at the beginning of the study, these events do not impact the analysis. The construction of the edentulism variable results in no missing data, as only those who answer yes or no are included in the study. Observations (unweighted) missing the baseline risk variables are 7 for activity limitations, 1 for perceived health status and 13 for smoking. Responses are imputed for all but one observation based on a future round of data. One observation was imputed as a non-smoker, so as not to remove their observation from the study. The entire cohort has 5,565 observations with 4,402 observations 18 and older. The final sample contains 4,245 observations representing 246,190,382 persons 18 and older (99% of the weighted 18+ US population).

Instead of analyzing the variables as separate inputs, clusters of similar baseline risk are created. Clusters are an effective way to uncover latent patterns and relationships, in contrast to regression methods. A weighted method of k-modes (wKModes in R) designed for inputs that are categorical was used. This method accounts for survey weighting and chooses the mode of each category on the basis of the sum of the weights. Frequency-based weighting was used, in contrast to simple matching, to examine the rarity of a category. If individuals shared a rare trait, they were considered more similar than if they shared a common trait. The algorithm was set with random starts and with convergence set at a maximum of 100 iterations, as is current practice. Finally, the algorithm was run 40 times, and the number of clusters was chosen on the basis of the lowest sum of squares.^18^

Seventeen clusters were chosen on the basis of the results from an elbow plot, a commonly used method.^19^^,^^20^ A larger number of clusters was chosen to allow for those within a cluster to be more similar, while creating clusters that differ more from one another.^21^ Finally, clusters were labeled by ranking the percentage of edentulism within the cluster from 1 (highest) to 17 (lowest).

To better describe and visualize the relationships among clusters, a second-stage clustering was performed to connect the clusters using hierarchical clustering with complete linkage.^22^ The results are represented by a dendrogram that visually resembles an inverted tree. The dendrogram is used to reduce the number of clusters into 3 larger blocks of clusters. In this way, the relationships among the clusters can be visualized, where the underlying baseline risk variables in the individual clusters are used to decide on the number of blocks.

Clusters that have a similar baseline risk were investigated and validated by comparing differences among the clusters by both the edentulous and death rates (and other external variables). Because the input variables were categorical, the distance measure chosen was cosine similarity.^23^ Cosine similarity measures pairwise correlation and has been used in text classification, genetics, and principal components.^24^, ^25^, ^26^, ^27^

## RESULTS

Initially, the components of the baseline risk of the clusters and their association with edentulism were described. Then the clusters were validated with the external variables to show the viability of the approach.

**Figure 1:**
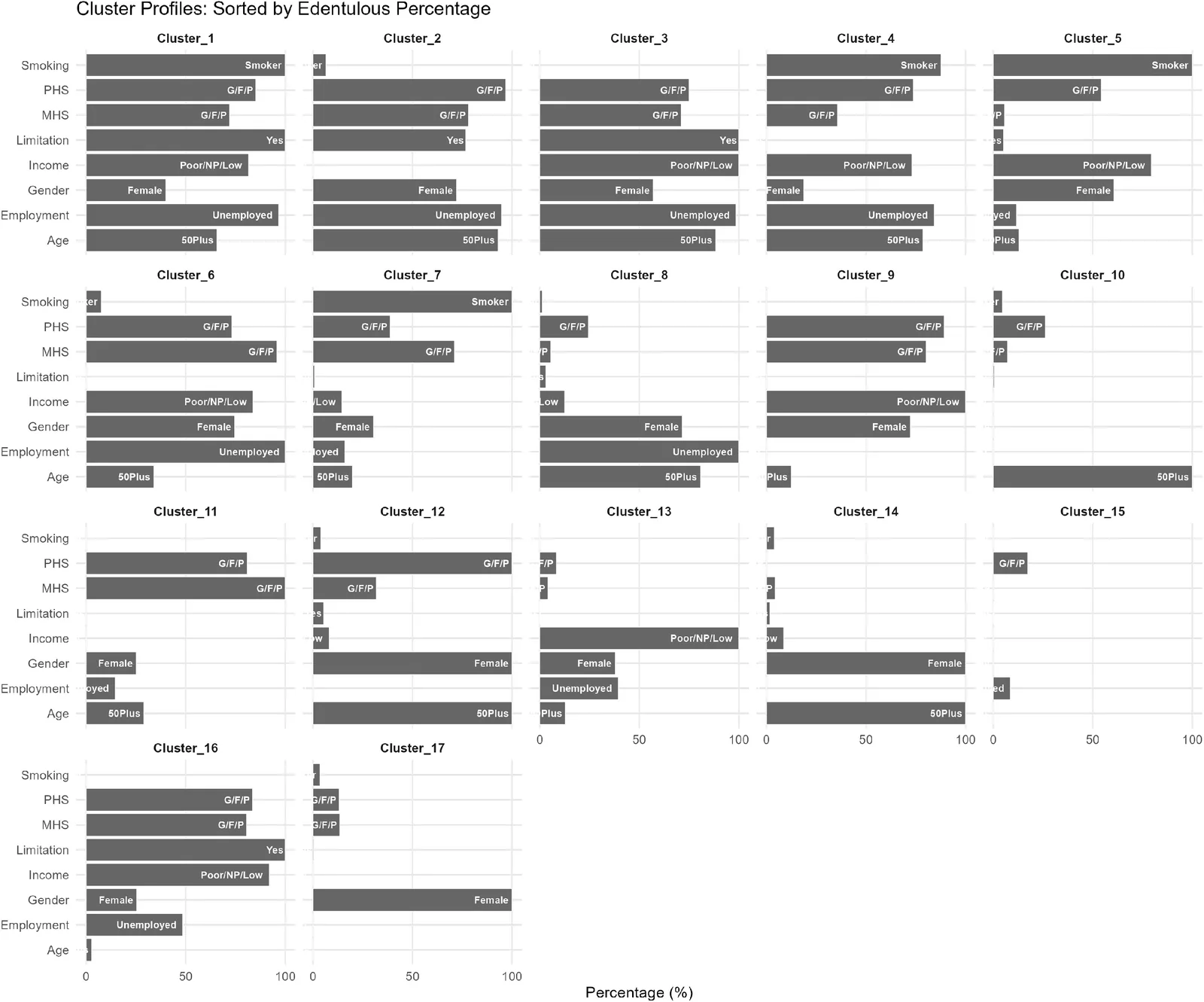
Summary of baseline risk, with clusters sorted from highest to lowest edentulous percentage. G/F/P, good/fair/poor; MHS, mental health status; PHS, perceived health status.

Overall, 11.9% of the cohort were edentulous, with 50.3% of Cluster 1 being edentulous and Clusters 2–4 having rates above 30%. Figure 1 helps to visualize the profiles of the clusters, with Appendix Table 1 (available online) showing the details. The clusters are labeled on the basis of the highest edentulism rate (Cluster 1) to the lowest (Cluster 17). Because all of the baseline variables are defined as binary, only 1 category per variable is needed. The percentage of each cluster is shown for smokers, those with good/fair/poor PHS or MHS, those with any limitation, those with poor/near poor/low income, females, the unemployed, and those aged ≥50 years. If the category is 50% or greater, it is considered the cluster mode. These collective categories are viewed as unfavorable risk factors.

Cluster 1 is formed by those who are smokers; those with good, fair, or poor PHS and MHS; those with limitations; those with poor, near poor, or low income; those unemployed for the first round in the study; and male aged >50 years. Those in the 17th cluster are described as nonsmokers, those with either excellent or very good PHS and MHS, those with no limitations, those with higher income, those employed, and female aged <50 years. Clusters 8, 15, and 17 are the largest clusters, with 13.5%, 13.9%, and 13.4% of the population, respectively. Cluster 16 is the smallest, with 0.7% of the population.

Looking across the clusters in Figure 1, the first 3 clusters are seen to have a greater number of unfavorable risk factors. Interestingly, the cluster with the highest edentulism rate is male dominated, and the second and third highest clusters for edentulism are nonsmoker dominated. Cluster 16 also has a higher number of unfavorable risk factors but has a low edentulism rate.

**Figure 2:**
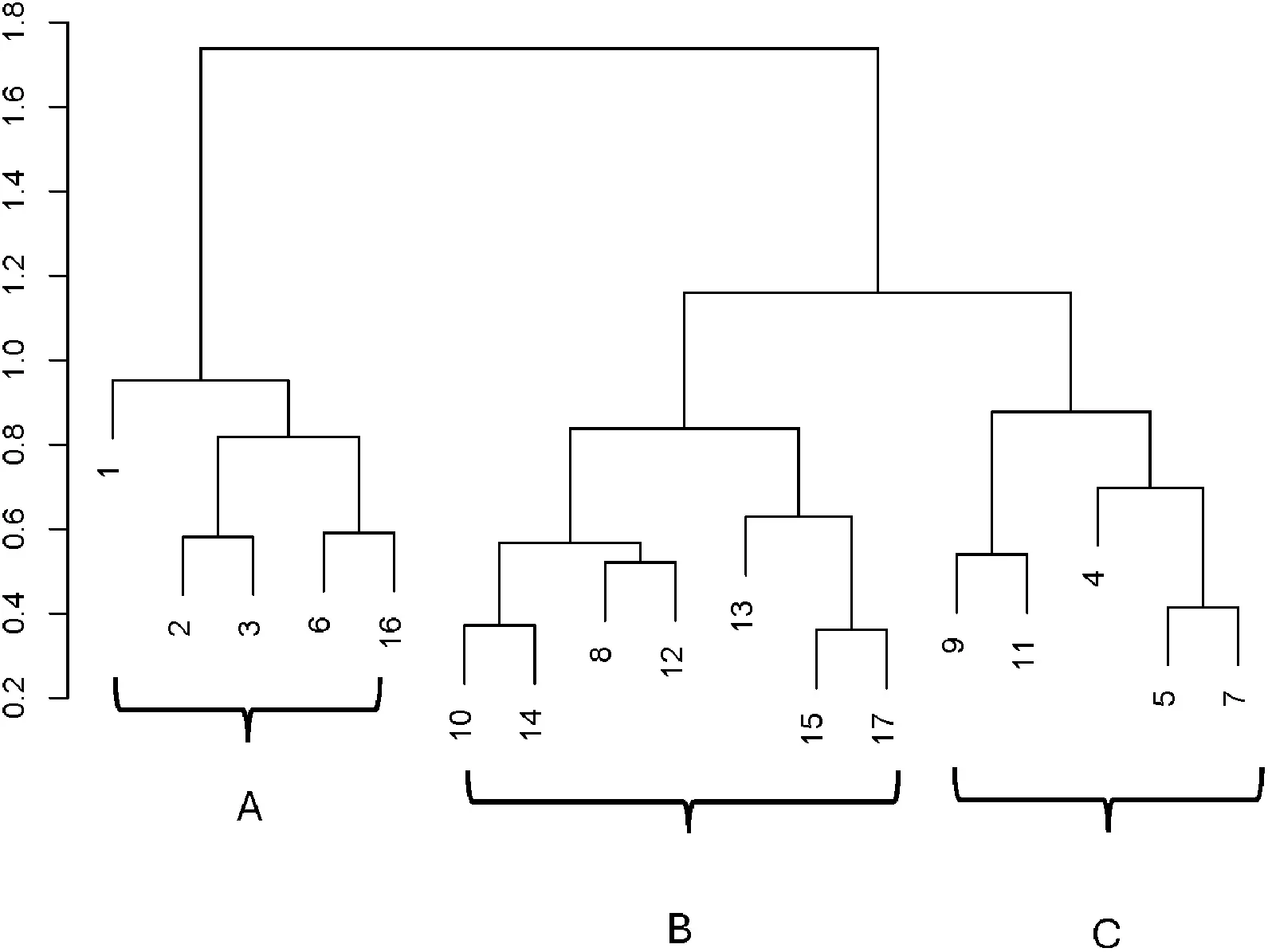
Dendrogram showing similarity among the 17 clusters.

These differences can be better visualized with a dendrogram output from the hierarchical clustering procedure shown in Figure 2. The height of the dendrogram reflects the distance between the clusters.^19^ Three blocks of clusters (A, B, and C) are defined as shown on the dendrogram.

**Figure 3:**
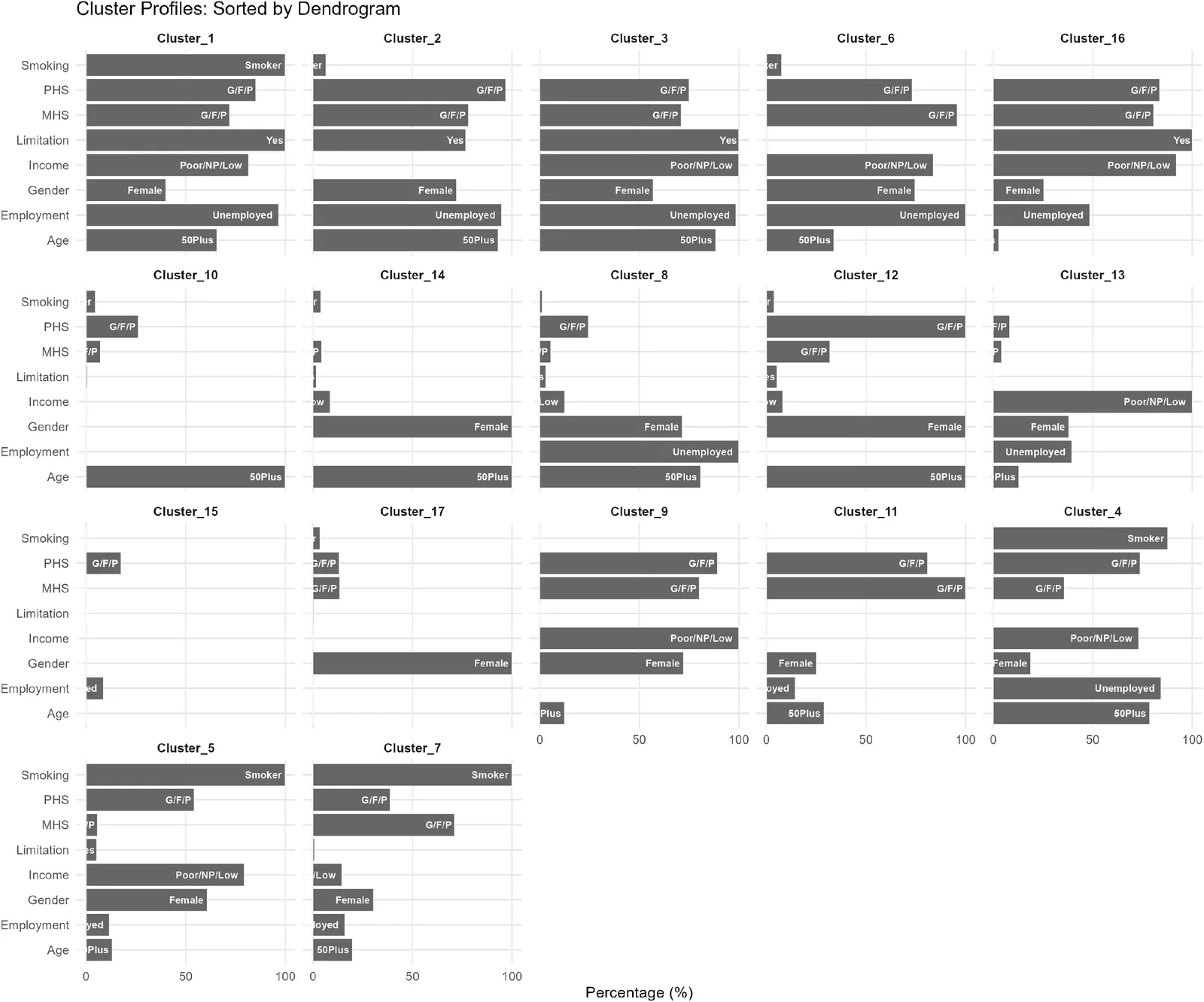
Summary of cluster percentages baseline risk, with clusters sorted by dendrogram output. G/F/P, good/fair/poor; MHS, mental health status; PHS, perceived health status.

This dendrogram can be better examined through a comparison within and across the blocks. Clusters 10 and 14 and Clusters 15 and 17 in Block B are the most similar pairs of clusters, because their paired distances are the smallest among all pairs of clusters. Clusters 2 and 3 in Block A are similar, as are Clusters 6 and 16, which are joined with Cluster 1. Clusters 4, 5, and 7 in Block C are most similar to one another. Block B has the least number of unfavorable risk factors, whereas Block A has the most. These similarities are more easily visualized by sorting the cluster output in the order of the dendrogram, as shown in Figure 3.

**Figure 4:**
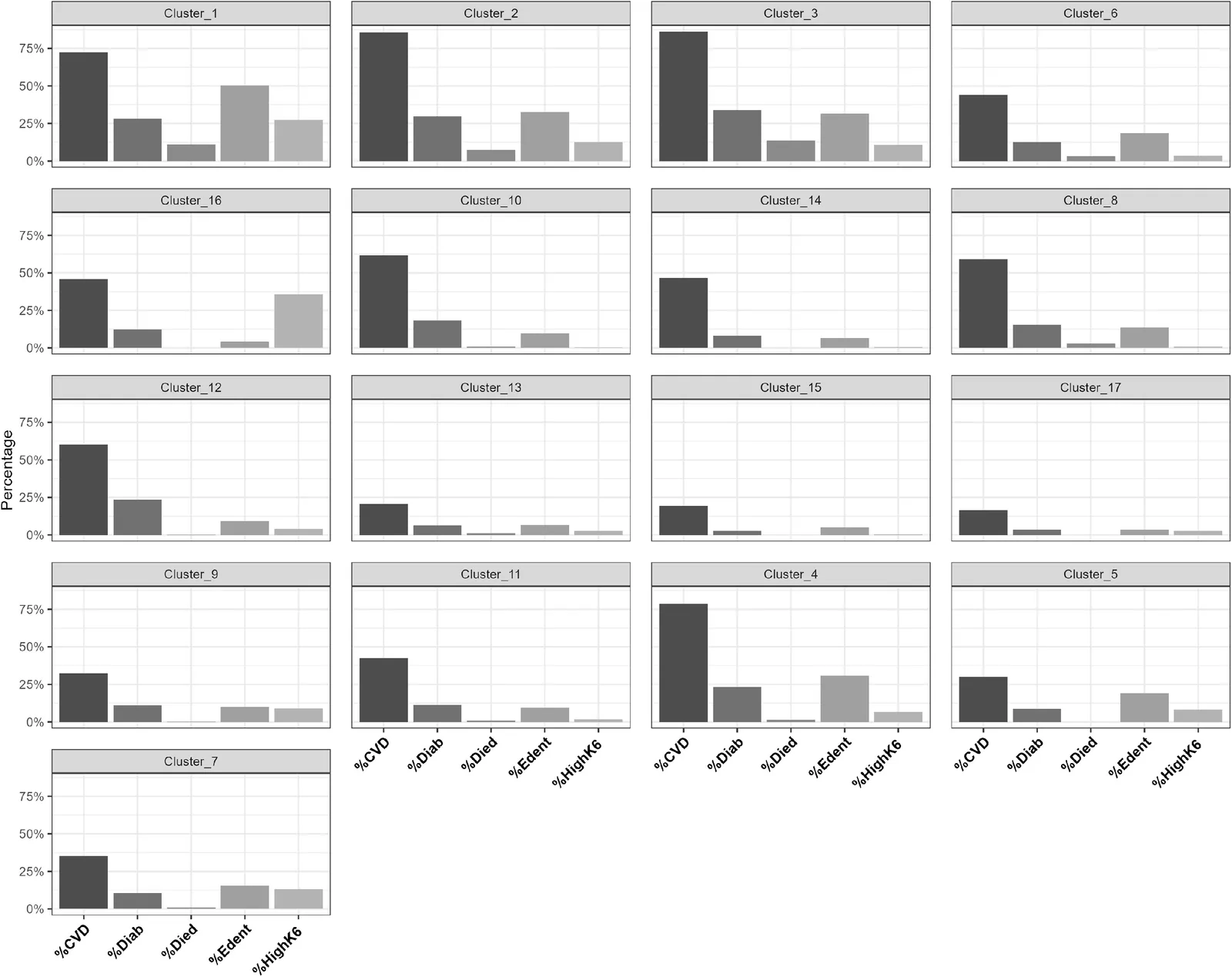
Summary of external variables by cluster, sorted by dendrogram output. CVD, cardiovascular disease.

The differences in external variables shown in Figure 4 are explored for clusters that are similar in their risk profile. Appendix Table 2 (available online) shows the details. First, differences between Clusters 1, 2, and 3 and Clusters 6 and 16 in the highest at-risk Block A are identified. Then, the differences between Clusters 9 and 11 and Clusters 4, 5, and 7 in Block C are also examined. Finally, the most similar pairs, 10 and 14, as well as 15 and 17, are in Block B of those least at risk.

Clusters 1, 2, and 3 have the highest edentulism rates as well as a high prevalence of other unfavorable risk factors, together with the worst outcomes. Overall, the 4-year death rate is 1.8%, yet Clusters 1 and 3 have death rates of 11.0% and 13.6%, respectively, the highest across all the clusters. Clusters 1–3 have the highest cluster percentages (over 28%) of those with diabetes. Percentages of those with CVD are among the highest in Clusters 1–4. Clusters 1–3 represent 8.4% of the population, accounting for 25.2% of the edentulous population and 50.0% of the deaths over the 4-year period measured for those who started in the study. These 3 clusters represent 21.7% of those with diabetes, 16.3% of those with CVD, and 33.7% of those with a high Kessler-6 score. Clusters 6 and 16 have a younger age distribution than Clusters 1, 2, and 3. Clusters 6 and 16’s external variables do not show the same level of poor outcomes as those in Clusters 1–3; however, the percentages of those with CVD are high in both clusters, and the high Kessler-6 score in Cluster 16 is the highest percentage across all the clusters.

Block C Clusters 9, 11, 4, 5, and 7 have fewer unfavorable risk factors than those in Block A. Clusters 9 and 11 are nonsmokers with a high percentage of individuals with risk factors for self-reported general health status and MHS, whereas Clusters 4, 5, and 7 are smokers but are mixed in the prevalence of those with high PHS and MHS. Clusters 5 and 7 are similar in the external variables, but Cluster 4 has a very high CVD cluster percentage of 78.6% as well as an older age distribution. The external variables for Clusters 9 and 11 are more similar to those for Clusters 5 and 7.

**Figure 5:**
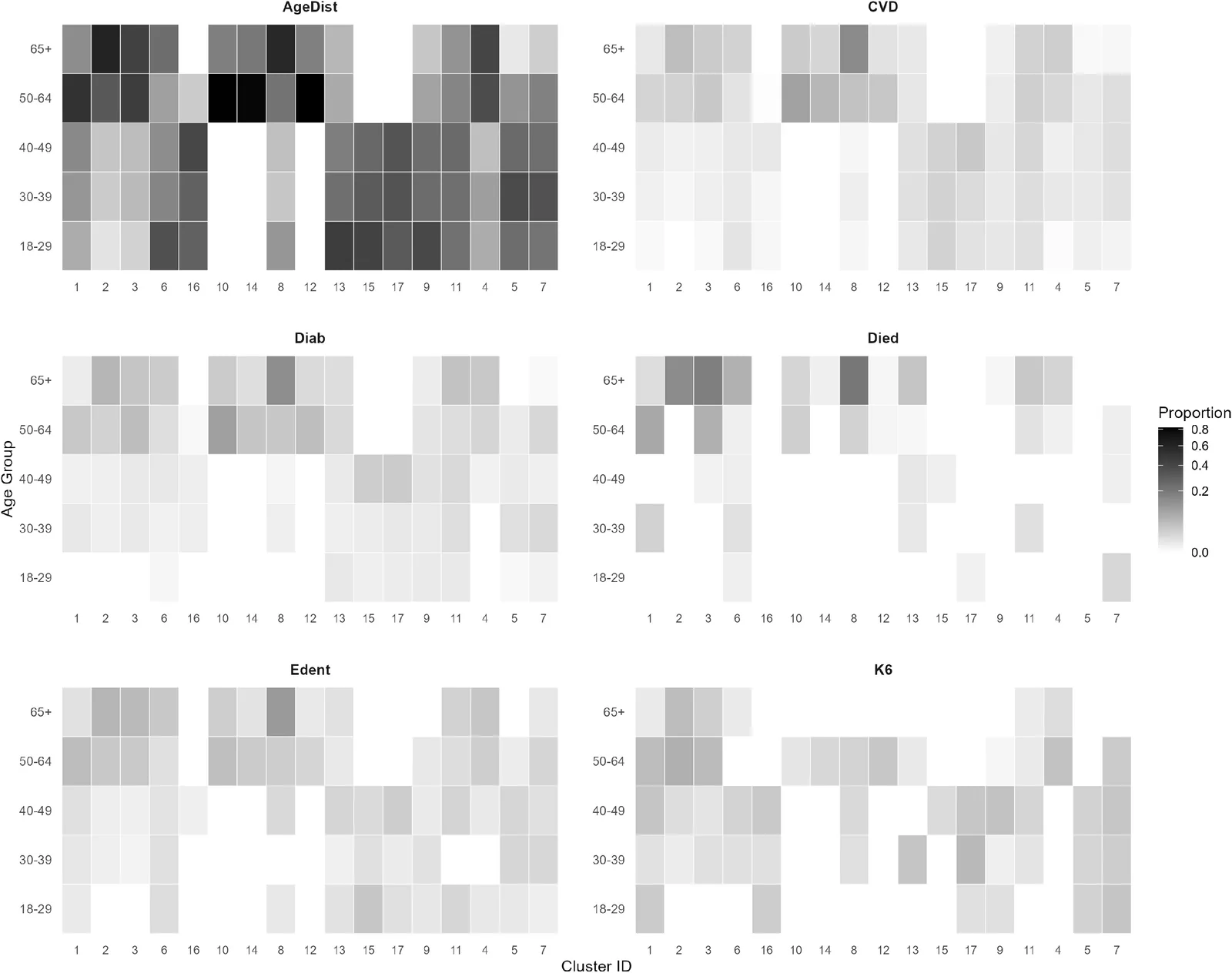
Summary of external variables by cluster and age group, sorted by dendrogram output. CVD, cardiovascular disease.

Clusters 10 and 14 in Block B mainly differ in the input variables, with Cluster 14 being entirely female, whereas Cluster 10 is entirely male. Their 4-year death rates and high Kessler-6 scores are low, but there is a greater percentage of those with diabetes and a much greater percentage of those with CVD in Cluster 10. The results for the external variables are very similar for Clusters 15 and 17, yet Cluster 15 is male, and Cluster 17 is female. Both of these clusters are skewed toward the younger age groups, in contrast to Clusters 10 and 14 (shown in Figure 5). Thus, although these individuals generally reported their health as excellent or very good, they have a greater prevalence of diagnosed conditions.

The discussion above indicates that age may be one reason why clusters with similar profiles may have different outcomes. Although the clusters are constructed with just a binary variable, Figure 5 shows detailed output by age category, sorted in the order of the dendrogram, with support in Appendix Table 3 (available online). The first panel is the distribution of age overall by cluster, with the columns summing to 1. Clusters 1–3 are skewed toward the higher age groups, whereas Clusters 6 and 16 are skewed toward the youngest age categories. Clusters 10 and 14 have distributions opposite to those of Clusters 15 and 17. Cluster 4 has the highest percentage of the oldest ages relative to Clusters 9, 11, 5, and 7.

The remaining 5 panels detail age by cluster by external variable (edentulism, death, CVD, diabetes, and high Kessler-6 scores). Each of the rows sums to the prevalence of the outcome by age category, the sums of the columns show the exposure by external variable, and the sum of all of the cells is 100%.

Although the presence of edentulism is associated with older ages, 34% of those aged <50 years are edentulous. The prevalence of edentulism at younger ages is noticeable. Similarly, although most deaths occur in the highest age groups, deaths in the 18–29 age group are present for Clusters 6, 17, and 7. CVD, diabetes, and high Kessler-6 scores are also present at many of the younger ages.

## DISCUSSION

This work provides a practical application to identifying at-risk groups for participating in impactful population health programs. Clusters create a joint profile of characteristics that reveal the relationship to edentulism, death, and other external variables. Clusters 1, 2, and 3 are the most at-risk clusters. Their numbers of baseline unfavorable risk factors are the highest as well as their links to edentulism and other external variables. From all of the clusters, profiles of individuals are identified using the modes of each risk factor. Clusters naturally consider the interactions of the input variables by grouping similar individuals together. The results reflect a combination of characteristics that affect outcomes, not individual characteristics. Traditional logistic models would need to include multilevel interaction terms to accomplish a similar result.

By ordering the clusters by the rate of edentulism, profiles of individuals with the highest edentulism rates can be compared. Clusters with similar profiles yet differing in their edentulism rates are also seen. The profiles in clusters with the highest rates of edentulism are not necessarily smokers, females, or of low income, as found in prior studies of edentulism using regression techniques. In fact, only 1 of the top 3 clusters for edentulism is smokers, and 2 of the 3 are female or of lower income. Perceived need, whether overall health status, MHS, or any limitations, is consistently high for these clusters.

The clusters are created on the basis of only the baseline risk variables and then labeled on the basis of the rate of edentulism, an external variable. One could easily have labeled the clusters by any of the other external variables. If that is done, Clusters 1–3 for edentulism are seen as the top 3 clusters for death and diabetes, among the top 4 for CVD, and among the top 5 for the Kessler-6 score. Thus, the most at-risk clusters are related across the external variables.

Clusters can be grouped together that are similar to one another in the baseline characteristics but could differ in the outcomes. CVD, diabetes, and high Kessler-6 scores are prevalent across age groups and present in the youngest age groups. In the cases described, the blocks contain younger individuals in clusters who are similar to those in other clusters with individuals who are older and have worse outcomes. These differences can be thought of as a way to forecast the future of these younger individuals. Programs could be designed to provide support for them as they age to help prevent edentulism, such as consistent and affordable preventive care. Dental care could be provided through school-based programs to help lower the onset of edentulism in later years. Adult dental programs could be designed at the state level and include preventive and restorative care. The most productive approach would be to include dental care with medical care. Access to and income support for restorative dental services, such as dentures, could improve the long-term health of those who are edentulous.^28^ For this study, approximately 30 million people in the U.S. (11.9% of the population) are estimated to be completely edentulous by summing the person-weights of those who are edentulous.

It is concerning that younger age groups are edentulous as well as showing a higher prevalence of CVD and high Kessler-6 scores in certain clusters. In particular, those in Cluster 16 could be targeted for integrated behavioral health programs.

Although the study period includes the coronavirus disease 2019 (COVID-19) years, edentulism is a condition that is years in the making and a marker of poor oral health. By utilizing the longer longitudinal data (4 years rather than 2 years for the MEPS design), a better measure of who is edentulous and the other external variables can be defined. The intent is not to explore any year-by-year changes in health but health over the total interval. An advantage of using self-reported general health status and MHS is to remove the need for a diagnosis from a provider and allow the individual to report how they feel. Thus, lack of access to a provider during the pandemic would not affect this measure. In addition, focusing on the self-reported measures alleviates the need to include variables such as insurance (either medical or dental insurance) and dental treatment itself, which was heavily impacted by the pandemic. The intent was to measure the individual’s health need and not the health care received. The use of the Kessler-6 score at Round 8 (2022) is useful for comparing post–COVID-19 mental health from the beginning of the study.

Clusters are a good tool for exploring the underlying relationships among a number of variables. In this study, the clusters are validated with external variables. However, in contrast to regression methods, there are choices in terms of the type of clustering method used and decisions on how to implement them. Because many of the clustering methods rely on random starts, one could get different results each time the procedure is run. To guard against spurious results, clusters are validated with external variables and other sensitivity analyses to ensure that the results, although not identical, arrive at similar conclusions.

### Limitations

There are some limitations to this study. The study is not a causal study. The MEPS data only reflect the non-institutional civilian population; those in nursing homes are excluded from the study if and when they enter an institution. Thus, the results in this study are understated as to the prevalence of edentulism among the total older population. The data are limited in that they do not measure all of the baseline variables and self-reported complete edentulism at the beginning of the study. There is no indication of how long an individual has been living with edentulism, when they became completely edentulous, or their functional dentition.

## CONCLUSIONS

This work has introduced a novel approach to study the collection of features that define baseline risk. The clustering methodology creates profiles of those who are at risk of being edentulous. An emphasis on population health initiatives that focus on affordable and preventive dental care at all ages will yield a high return on investment. Providing targeted interventions to those who smoke, as in Clusters 4, 5, and 7, could help prevent future edentulism. Providing targeted interventions to those in cluster Block A could improve their health. Identifying those clusters with a propensity to develop higher edentulism rates could focus on education to improve their oral health literacy and the importance of consistent dental visits to help ensure stable future oral health and overall health.^29^, ^30^, ^31^, ^32^

## Appendix Supplementary materials

Image, application 1

## Footnotes

Supplementary material associated with this article can be found in the online version at doi:10.1016/j.focus.2026.100505.
