# Supplementary material for: Identifying High-Priority Profiles for Edentulism and Other Health Outcomes Through Population-Based Clustering
Source: AJPM Focus. 2026 May 1;5(5):100505. doi: 10.1016/j.focus.2026.100505 (PMC13499523; doi:10.1016/j.focus.2026.100505)
Supplement: Supplementary file 1 [file mmc1.pdf]

Identifying High-Priority Profiles for Edentulism and Other Health Outcomes Through Population-Based Clustering

Marjorie A. Rosenberg, PhD, FSA
University of Wisconsin-Madison
margie.rosenberg@wisc.edu

AJPM Focus

Table A1: Summary of Cluster Percentages Sorted by Highest to Lowest Edentulous Percentage for Baseline Risk Input Variables

	**Overall**	**1**	**2**	**3**	**4**	**5**	**6**	**7**	**8**	**9**	**10**	**11**	**12**	**13**	**14**	**15**	**16**	**17**
**% Pop by Cluster**	100	1.8%	3.4%	3.2%	2.6%	2.6%	4.5%	4.0%	13.5%	3.1%	8.2%	8.3%	2.9%	7.5%	6.6%	13.9%	0.7%	13.4%
**% Edentulous by Cluster**	11.9%	50.3%	32.6%	31.6%	30.8%	19.2%	18.5%	15.6%	13.6%	10.0%	9.8%	9.7%	9.2%	6.7%	6.6%	5.0%	4.2%	3.5%
**% Died by Cluster**	1.8%	11.0%	7.5%	13.6%	1.4%	0.0%	3.2%	0.9%	3.0%	0.1%	0.9%	1.0%	0.1%	1.1%	0.1%	0.0%	0.0%	0.0%
**Age**																		
18-49	55.9%	34.3%	7.0%	11.6%	21.5%	**87.0%**	**66.1%**	**80.2%**	19.2%	**87.6%**	0.0%	**71.1%**	0.0%	**87.1%**	0.0%	**100.0%**	**97.2%**	**100.0%**
50+	44.1%	**65.7%**	**93.0%**	**88.4%**	**78.5%**	13.0%	33.9%	19.8%	80.8%	12.4%	**100.0%**	28.9%	**100.0%**	12.9%	**100.0%**	0.0%	2.8%	0.0%
**Gender**																		
Male	48.6%	**60.1%**	27.9%	43.0%	**81.2%**	39.3%	25.4%	**69.6%**	28.4%	27.8%	**100.0%**	**74.9%**	0.0%	**62.1%**	0.0%	**100.0%**	**74.6%**	0.0%
Female	51.4%	39.9%	**72.1%**	**57.0%**	18.8%	**60.7%**	**74.6%**	30.4%	**71.6%**	**72.2%**	0.0%	25.1%	**100.0%**	37.9%	**100.0%**	0.0%	25.4%	**100.0%**
**Smoking Status**																		
Non-Smoker	87.3%	0.0%	**93.5%**	**100.0%**	12.3%	0.0%	**92.4%**	0.0%	**98.7%**	**100.0%**	**95.4%**	**100.0%**	**96.1%**	**100.0%**	**96.0%**	**100.0%**	**100.0%**	**96.5%**
Smoker	12.7%	**100.0%**	6.5%	0.0%	**87.7%**	**100.0%**	7.6%	**100.0%**	1.3%	0.0%	4.6%	0.0%	3.9%	0.0%	4.0%	0.0%	0.0%	3.5%
**Perceived Health Status**																		
E/VG	61.5%	14.8%	3.3%	25.0%	26.3%	45.8%	26.8%	**61.2%**	**75.6%**	10.7%	**73.9%**	19.1%	0.0%	**91.7%**	**100.0%**	**82.6%**	16.4%	**86.8%**
G/F/P	38.5%	**85.2%**	**96.7%**	**75.0%**	**73.7%**	**54.2%**	**73.2%**	38.8%	24.4%	**89.3%**	26.1%	**80.9%**	**100.0%**	8.3%	0.0%	17.4%	**83.6%**	13.2%
**Mental Health Status**																		
E/VG	69.6%	28.0%	21.9%	29.0%	**64.3%**	**94.3%**	4.2%	29.0%	**94.4%**	19.9%	**92.8%**	0.0%	**68.2%**	**95.8%**	**95.6%**	**100.0%**	19.4%	**86.5%**
G/F/P	30.4%	**72.0%**	**78.1%**	**71.0%**	35.7%	5.7%	**95.8%**	**71.0%**	5.6%	**80.1%**	7.2%	**100.0%**	31.8%	4.2%	4.4%	0.0%	**80.6%**	13.5%
**Any Limitation**																		
No	90.8%	0.0%	23.3%	0.0%	**100.0%**	**94.8%**	**100.0%**	**99.3%**	**96.9%**	**100.0%**	**99.5%**	**100.0%**	**94.7%**	**99.7%**	**98.3%**	**99.8%**	0.0%	**99.6%**
Yes	9.2%	**100.0%**	**76.7%**	**100.0%**	0.0%	5.2%	0.0%	0.7%	3.1%	0.0%	0.5%	0.0%	5.3%	0.3%	1.7%	0.2%	**100.0%**	0.4%
**Income Category**																		
Middle/High	73.3%	18.5%	**100.0%**	0.0%	26.9%	20.6%	16.2%	**85.4%**	**87.5%**	0.0%	**100.0%**	**100.0%**	**91.9%**	0.0%	**91.3%**	**100.0%**	8.1%	**100.0%**
Poor/NP/Low	26.7%	**81.5%**	0.0%	**100.0%**	**73.1%**	**79.4%**	**83.8%**	14.6%	12.5%	**100.0%**	0.0%	0.0%	8.1%	**100.0%**	8.7%	0.0%	**91.9%**	0.0%
**Employment**																		
Employed	65.1%	3.4%	5.3%	1.4%	15.8%	**88.3%**	0.0%	**84.0%**	0.0%	**100.0%**	**100.0%**	**85.5%**	**100.0%**	**60.6%**	**100.0%**	**91.4%**	**51.6%**	**100.0%**
Unemployed	34.9%	**96.6%**	**94.7%**	**98.6%**	**84.2%**	11.7%	**100.0%**	16.0%	**100.0%**	0.0%	0.0%	14.5%	0.0%	39.4%	0.0%	8.6%	48.4%	0.0%

Table A2: Summary of Cluster Percentages Sorted by Highest to Lowest Edentulous Percentage for External Variables

	Overall	1	2	3	4	5	6	7	8	9	10	11	12	13	14	15	16	17
**% Pop by Cluster**	100	1.8%	3.4%	3.2%	2.6%	2.6%	4.5%	4.0%	13.5%	3.1%	8.2%	8.3%	2.9%	7.5%	6.6%	13.9%	0.7%	13.4%
Raw Numbers	4245	117	191	274	151	116	294	144	616	171	314	302	134	327	274	389	35	396
Survey-Adjusted Numbers	246190382	4398812	8253867	7842288	6406870	6515022	11048244	9791978	33231744	7566122	20226746	20525929	7061297	18418657	16136932	34124007	1688973	32952894
**% Edentulous by Cluster**	11.9%	50.3%	32.6%	31.6%	30.8%	19.2%	18.5%	15.6%	13.6%	10.0%	9.8%	9.7%	9.2%	6.7%	6.6%	5.0%	4.2%	3.5%
95% Confidence Interval	(0.104, 0.134)	(0.356, 0.650)	(0.236, 0.415)	(0.224, 0.408)	(0.208, 0.409)	(0.106, 0.279)	(0.120, 0.25)	(0.080, 0.231)	(0.094, 0.177)	(0.037, 0.163)	(0.05, 0.145)	(0.047, 0.147)	(0.039, 0.145)	(0.031, 0.103)	(0.022, 0.111)	(0.025, 0.074)	(0, 0.106)	(0.013, 0.056)
**% Died by Cluster**	1.8%	11.0%	7.5%	13.6%	1.4%	0.0%	3.2%	0.9%	3.0%	0.1%	0.9%	1.0%	0.1%	1.1%	0.1%	0.0%	0.0%	0.0%
95% Confidence Interval	(0.0133, 0.022)	(0.018, 0.202)	(0.028, 0.122)	(0.059, 0.213)	(0.002, 0.027)	(0, 0)	(0.012, 0.053)	(0, 0.024)	(0.014, 0.045)	(0, 0.002)	(0.001, 0.017)	(0, 0.022)	(0, 0.003)	(0.001, 0.021)	(0, 0.002)	(0, 0.001)	(0, 0)	(0, 0.001)
**Diabetes**	11.9%	28.1%	29.6%	33.8%	23.2%	8.6%	12.5%	10.6%	15.4%	11.2%	18.3%	11.4%	23.5%	6.3%	8.1%	2.8%	12.2%	3.4%
95% Confidence Interval	(0.105, 0.132)	(0.186, 0.377)	(0.201, 0.391)	(0.251, 0.426)	(0.114, 0.35)	(0.013, 0.159)	(0.079, 0.171	(0.023, 0.188)	(0.121, 0.188)	(0.063, 0.16)	(0.118, 0.248)	(0.075, 0.153)	(0.144, 0.325)	(0.036, 0.091)	(0.041, 0.121)	(0.009, 0.047)	).015, 0.229	(0.018, 0.05)
**CVD**	42.3%	72.6%	85.6%	86.1%	78.6%	30.1%	44.1%	35.3%	59.0%	32.3%	61.7%	42.5%	60.1%	20.8%	46.6%	19.4%	45.8%	16.5%
95% Confidence Interval	(0.400, 0.446)	(0.611, 0.84)	(0.784, 0.927)	(0.796, 0.925)	(0.687, 0.884)	(0.192, 0.411)	(0.352, 0.53)	(0.247, 0.458)	(0.536, 0.645)	(0.221, 0.424)	(0.537, 0.697)	(0.348, 0.503)	(0.484, 0.718)	(0.152, 0.264)	(0.395, 0.538)	(0.143, 0.244)	0.243, 0.674	(0.123, 0.208)
**K6 Round 8 (High)**	3.9%	27.3%	12.6%	10.8%	6.7%	8.2%	3.7%	13.1%	1.0%	9.0%	0.3%	1.7%	4.1%	2.8%	0.8%	0.5%	35.8%	2.8%
95% Confidence Interval	(0.056, 0.085)	(0.139, 0.448)	(0.132, 0.332)	(0.15, 0.314)	(0.07, 0.254)	(0.037, 0.201)	(0.055, 0.14)	(0.031, 0.241)	(0.015, 0.068)	(0.047, 0.218)	(0.008, 0.07)	(0.013, 0.084)	(-0.004, 0.022)	(0.004, 0.09)	(-0.007, 0.025)	(-0.001, 0.033)	0.154, 0.738	(0.008, 0.073)

Table A3: Summary of Cluster Percentages Sorted by Highest to Lowest Edentulous Percentage for External Variables by Age Group

	Overall	1	2	3	4	5	6	7	8	9	10	11	12	13	14	15	16	17
**% Pop by Cluster**	100	1.8%	3.4%	3.2%	2.6%	2.6%	4.5%	4.0%	13.5%	3.1%	8.2%	8.3%	2.9%	7.5%	6.6%	13.9%	0.7%	13.4%
**Age**																		
18-29	22.0%	7.2%	0.8%	2.1%	7.3%	23.9%	36.0%	21.6%	11.8%	39.5%	0.0%	22.5%	0.0%	45.7%	0.0%	42.9%	28.7%	31.5%
30-39	17.9%	11.9%	2.7%	4.9%	10.0%	38.2%	16.2%	35.1%	3.4%	24.3%	0.0%	23.4%	0.0%	23.1%	0.0%	31.0%	28.4%	34.7%
40-49	16.0%	15.1%	3.5%	4.7%	4.2%	24.9%	14.0%	23.6%	4.1%	23.9%	0.0%	25.1%	0.0%	18.4%	0.0%	26.1%	40.1%	33.8%
50-64	25.4%	51.6%	31.8%	45.3%	37.6%	12.4%	9.8%	17.3%	21.9%	9.0%	82.0%	16.3%	82.3%	7.5%	79.9%	0.0%	2.8%	0.0%
65+	18.7%	14.1%	61.2%	43.1%	40.9%	0.5%	24.1%	2.5%	58.9%	3.3%	18.0%	12.6%	17.7%	5.3%	20.1%	0.0%	0.0%	0.0%
**Those who are Edentulous By Age**																		
18-29	11.0%	0.43%	0.00%	0.00%	0.51%	0.54%	1.16%	0.31%	0.55%	0.64%	0.00%	1.72%	0.00%	0.85%	0.00%	3.39%	0.00%	0.94%
30-39	7.9%	0.62%	0.29%	0.13%	0.00%	1.68%	1.03%	1.61%	0.00%	0.91%	0.00%	0.00%	0.00%	0.21%	0.00%	1.02%	0.00%	0.41%
40-49	15.4%	0.99%	0.33%	0.29%	0.49%	1.70%	0.78%	0.98%	1.49%	0.44%	0.00%	1.90%	0.00%	1.76%	0.00%	1.41%	0.24%	2.55%
50-64	30.0%	4.64%	3.08%	3.04%	2.45%	0.36%	0.97%	1.79%	2.56%	0.59%	4.25%	1.13%	1.75%	0.47%	2.86%	0.00%	0.00%	0.00%
65+	35.8%	0.88%	5.48%	5.00%	3.30%	0.00%	3.06%	0.52%	10.82%	0.00%	2.49%	2.02%	0.47%	0.90%	0.80%	0.00%	0.00%	0.00%
**Those who Died By Age**																		
18-29	2.1%	0.00%	0.00%	0.00%	0.00%	0.00%	0.30%	1.58%	0.00%	0.00%	0.00%	0.00%	0.00%	0.00%	0.00%	0.00%	0.00%	0.19%
30-39	4.5%	2.07%	0.00%	0.00%	0.00%	0.00%	0.89%	0.00%	0.00%	0.00%	0.00%	0.97%	0.00%	0.57%	0.00%	0.00%	0.00%	0.00%
40-49	1.6%	0.00%	0.00%	0.16%	0.00%	0.00%	0.31%	0.25%	0.00%	0.00%	0.00%	0.00%	0.00%	0.63%	0.00%	0.29%	0.00%	0.00%
50-64	20.8%	7.94%	0.00%	6.46%	0.23%	0.00%	0.27%	0.31%	2.18%	0.00%	2.34%	0.85%	0.13%	0.07%	0.00%	0.00%	0.00%	0.00%
65+	71.0%	1.14%	14.23%	17.98%	1.92%	0.00%	6.41%	0.00%	20.49%	0.10%	1.83%	3.09%	0.10%	3.43%	0.30%	0.00%	0.00%	0.00%
**Those who Have CVD By Age**																		
18-29	6.5%	0.04%	0.00%	0.04%	0.01%	0.26%	0.76%	0.15%	0.08%	0.64%	0.00%	0.98%	0.00%	0.53%	0.00%	2.09%	0.06%	0.85%
30-39	9.5%	0.24%	0.08%	0.28%	0.55%	0.49%	0.47%	0.88%	0.32%	0.49%	0.00%	1.19%	0.00%	0.96%	0.00%	2.19%	0.08%	1.33%
40-49	12.4%	0.38%	0.19%	0.24%	0.25%	0.55%	0.54%	1.14%	0.11%	0.64%	0.00%	1.67%	0.00%	0.98%	0.00%	2.08%	0.60%	3.06%
50-64	36.1%	1.85%	2.19%	3.12%	1.60%	0.55%	0.78%	1.10%	3.84%	0.35%	9.08%	2.38%	3.23%	0.62%	5.43%	0.00%	0.01%	0.00%
65+	35.4%	0.56%	4.32%	2.81%	2.43%	0.03%	2.14%	0.06%	14.51%	0.22%	2.92%	2.16%	0.84%	0.60%	1.80%	0.00%	0.00%	0.00%
**Those who Have Diabetes By Age**																		
18-29	2.5%	0.00%	0.00%	0.00%	0.00%	0.05%	0.10%	0.17%	0.00%	0.46%	0.00%	0.48%	0.00%	0.57%	0.00%	0.30%	0.00%	0.36%
30-39	7.3%	0.57%	0.29%	0.49%	0.00%	1.05%	0.21%	1.43%	0.27%	0.45%	0.00%	1.07%	0.00%	0.26%	0.00%	0.37%	0.32%	0.57%
40-49	11.2%	0.22%	0.26%	0.47%	0.20%	0.42%	0.57%	0.30%	0.11%	0.90%	0.00%	1.50%	0.00%	0.45%	0.00%	2.59%	0.34%	2.88%
50-64	39.0%	3.07%	2.16%	4.62%	1.77%	0.40%	1.09%	1.60%	3.26%	0.71%	9.87%	1.17%	4.32%	1.53%	3.41%	0.00%	0.04%	0.00%
65+	39.9%	0.38%	5.66%	3.51%	3.12%	0.00%	2.76%	0.04%	13.92%	0.37%	2.78%	3.79%	1.35%	1.17%	1.06%	0.00%	0.00%	0.00%
**Those with High K6 Scores By Age**																		
18-29	13.0%	2.80%	0.00%	0.00%	0.00%	2.08%	0.00%	3.49%	0.00%	1.03%	0.00%	0.00%	0.00%	0.00%	0.00%	0.00%	2.56%	1.02%
30-39	19.1%	0.91%	0.37%	0.95%	0.00%	1.77%	0.98%	2.51%	1.05%	0.26%	0.00%	0.75%	0.00%	3.43%	0.00%	0.00%	0.94%	5.12%
40-49	27.5%	3.49%	1.15%	0.73%	0.00%	2.11%	1.94%	3.38%	1.45%	3.88%	0.00%	1.80%	0.00%	0.00%	0.00%	1.28%	3.01%	3.30%
50-64	31.1%	4.59%	6.57%	4.78%	3.81%	0.00%	0.00%	2.91%	1.93%	0.08%	0.67%	0.43%	3.21%	0.47%	1.65%	0.00%	0.00%	0.00%
65+	9.4%	0.44%	4.53%	2.35%	1.18%	0.00%	0.44%	0.00%	0.00%	0.00%	0.00%	0.41%	0.00%	0.00%	0.00%	0.00%	0.00%	0.00%

The top block shows the age distribution overall and by cluster. The bottom blocks show the percentage of the edentulous population by age category, with the rows by cluster adding up to the percentage by age category.
